# Overexpression of Angiopoietin-1 Increases CD133+/c-kit+ Cells and Reduces Myocardial Apoptosis in db/db Mouse Infarcted Hearts

**DOI:** 10.1371/journal.pone.0035905

**Published:** 2012-04-27

**Authors:** Heng Zeng, Lanfang Li, Jian-Xiong Chen

**Affiliations:** Department of Pharmacology and Toxicology, University of Mississippi Medical Center, Jackson, Mississippi, United States of America; University of Pecs Medical School, Hungary

## Abstract

Hematopoietic progenitor CD133^+^/c-kit^+^ cells have been shown to be involved in myocardial healing following myocardial infarction (MI). Previously we demonstrated that angiopoietin-1(Ang-1) is beneficial in the repair of diabetic infarcted hearts. We now investigate whether Ang-1 affects CD133^+^/c-kit^+^ cell recruitment to the infarcted myocardium thereby mediating cardiac repair in type II (db/db) diabetic mice. db/db mice were administered either adenovirus Ang-1 (Ad-Ang-1) or Ad-β-gal systemically immediately after ligation of the left anterior descending coronary artery (LAD). Overexpression of Ang-1 resulted in a significant increase in CXCR-4/SDF-1α expression and promoted CD133^+^/c-kit^+^, CD133^+^/CXCR-4^+^ and CD133^+^/SDF-1α^+^ cell recruitment into ischemic hearts. Overexpression of Ang-1 led to significant increases in number of CD31^+^ and smooth muscle-like cells and VEGF expression in bone marrow (BM). This was accompanied by significant decreases in cardiac apoptosis and fibrosis and an increase in myocardial capillary density. Ang-1 also upregulated Jagged-1, Notch3 and apelin expression followed by increases in arteriole formation in the infarcted myocardium. Furthermore, overexpression of Ang-1 resulted in a significant improvement of cardiac functional recovery after 14 days of ischemia. Our data strongly suggest that Ang-1 attenuates cardiac apoptosis and promotes cardiac repair by a mechanism involving in promoting CD133^+^/c-kit^+^ cells and angiogenesis in diabetic db/db mouse infarcted hearts.

## Introduction

Ang-1 is an oligomeric-secreted glycoprotein, which binds to Tie-2 and induces Tie-2 phosphorylation. Ang-1 is recognized as a survival factor for endothelial cells (EC). Treatment with Ang-1 prevents EC apoptosis via activation of the PI3K/Akt pathway.[1], [2] Ang-1 has also been shown to prevent diabetic retinopathy by attenuating retinal permeability in the streptozotocin (STZ)-induced rat diabetic model.[3] Our previous studies revealed that overexpression of Ang-1 in diabetic db/db mouse heart restores Tie-2 expression and significantly increases myocardial capillary formation; this is accompanied by a dramatic decrease in myocardial hypertrophy and cardiac fibrosis.[4] These data implicate Ang-1 as a potential therapeutic target in the treatment of diabetic cardiovascular complications.

Endothelial progenitor cells (EPCs) home to sites of ischemia and contribute to neovascularization in ischemic tissue.[5] Experimental and clinical studies demonstrate that treatment of acute myocardial infarction with EPCs results in a reduction in infarct size.[6], [7] Vascular progenitor cells have been shown to differentiate into cardiomyocytes and vascular smooth muscle cells (VSMC), which may contribute to cardiac and/or vascular regeneration following myocardial infarction [8], [9]. Intriguingly, the differentiation of EPCs is impaired in both diabetic patients with coronary artery disease and in diabetic mouse models [10], [11]. Previously we demonstrate that the level of EPCs is significantly decreased in STZ-induced diabetic mouse compared to non-diabetic mice [12]. Our previous studies also reveal that disruption of BM-EPC differentiation and impairment of angiogenesis after myocardial ischemia are associated with larger myocardial infarct size in the diabetic STZ mice [12]. These studies suggest that impaired vascular progenitor cell recruitment and failure of BM differentiation to EPCs after MI may contribute to insufficient angiogenesis and exacerbation of MI in diabetes. Thus, an agent that promotes vascular progenitor cell recruitment and angiogenesis will be beneficial for ischemic injury repair and cardiac remodeling after MI in diabetic hearts. This notion is supported by our previous work demonstrating that overexpression of Ang-1 significantly increased myocardial angiogenesis and reduced myocardial infarction size in the STZ diabetic mouse model [12]. However, the underlying molecular mechanism by which Ang-1 attenuates myocardial ischemic injury in the diabetic heart following MI remains poorly understood.

Ang-1 has been shown to have a critical role in the maintenance of hematopoietic stem cell in the bone marrow through its binding to the Tie-2 receptor.[13]The hematopoietic stem cell cytokine SDF-1α and it receptor CXCR-4 have been identified as the central signaling axis that regulates recruitment of hematopoietic stem cells into the injured area of myocardial ischemia and in improvement of cardiac function after MI [14]. Using diabetic db/db mice subjected to myocardial ischemia, the present study investigates whether overexpression of Ang-1 promotes recruitment of hematopoietic progenitor cells into ischemic sites and whether this leads to attenuation of myocardial ischemic injury through SDF-1α/CXCR-4 signaling. Our data suggest that Ang-1/Tie-2 plays a crucial role in regulation of hematopoietic progenitor cell recruitment and cardiac repair in the diabetic infarcted heart.

## Methods

### Ethics Statement

All procedures conformed to the Institute for Laboratory Animal Research Guide for the Care and Use of Laboratory Animals and were approved by the University of Mississippi Medical Center Institutional Animal Care and Use Committee (Protocol ID: #1280).

### Diabetic mouse myocardial ischemia model

db/db mice (12–14 weeks of age) were purchased from Jackson Laboratory (Bar Harbor, Maine). Experimental mice were anesthetized with ketamine (100 mg/kg) plus xylazine (15 mg/kg), intubated and artificially ventilated with room air. Adequate anesthesia was monitored by toe pinch. A left thoracotomy was performed and myocardial ischemia was achieved by ligation of the left anterior descending coronary artery (LAD) [12], [15]. Sham controls underwent the surgery without LAD ligation. Experimental mice were divided into three groups: (1) sham control (n = 12 mice); (2) Ad-β-gal + ischemia 24 hours (n = 22 mice) and 14 days (n = 13 mice) and (3) Ad-Ang-1 + ischemia 24 hours (n = 16 mice) and 14 days (n = 19 mice). Experimental mice were sacrificed under anesthesia with isoflurane at 24 hours and 14 days after LAD ligation for the analyses described below.

### Systemic delivery of Ang-1 to experimental mice

After surgery, db/db mice received an intravenous tail vein injection of either Ad-Ang-1 (1×10^9^ PFU) or Ad-β-gal (1×10^9^ PFU) 4,12.

### Western analysis of Ang-1, SDF-1α, VEGF, apelin, APLNR (APJ), Jagged 1 and Notch3 expression

After 24 hours of myocardial ischemia, the hearts and BM were harvested and homogenized in lysis buffer. The membranes were immunoblotted with SDF-1α, VEGF, apelin, APNLR, Jagged1 and Notch3 (1∶1000, Santa Cruz, CA) or Ang-1 (1∶1000, Sigma, MO) antibodies. The membranes were washed and incubated with a secondary antibody coupled to horseradish peroxidase and densitometric analysis was carried out using image acquisition and analysis software (TINA 2.0).

### Myocardial Ang-1, Tie-2, CD133, CD45, C-kit, CXCR-4, SDF-1α, SMA and Notch3 expression

Heart tissue sections (8 µm thick) were incubated with Ang-1, CD133, Tie-2 and Notch3, CXCR-4, CD45, c-kit and SDF-1α (1∶200 Santa Cruz, CA) antibodies overnight. Ang-1, CD133, Tie-2 and Notch3 were visualized using FITC labeled goat anti-mouse IgG antibodies; CXCR-4, CD45 and SDF-1α were visualized with Fluorolink^TM^ Cy^TM^3 labeled goat anti-mouse IgG antibodies (1∶200). Cy3-conjugated anti-α smooth muscle actin (SMA, 1∶100; Sigma, MO) was used to identify smooth muscle cells. Myocardial CD133, CD45 and CXCR-4 expression was assessed by counting the number of positive cells per square millimeter (mm^2^) of tissue. Co-localization of CD133 with either CXCR-4 or SDF-1α, or Ang-1 with SMA was visualized with a fluorescence microscope (Nikon TE 2000, Japan). Sections were counterstained with DAPI.

### Bone marrow (BM) cell differentiation into CD31^+^ and SMA^+^ cells

At 24 hours of myocardial ischemia, BM cells were obtained by flushing the tibias and femurs with 10% FBS EGM. Immediately after isolation, 10^5^ bone marrow–derived mononuclear cells were plated on 6-well culture plates coated with fibronectin (50 μg/ml, Sigma). After 4 days of culture, the nonadherent cells were removed and the adherent cells were incubated with -FITC-labeled CD31 antibody (1∶100, BD Biosciences) and -Cy3-conjugated anti-α smooth muscle actin (SMA, 1∶100; Sigma, MO). Positively-stained cells were counted per 10^5^ BM cells.

### Analysis of myocardial capillary density

After 14 days of myocardial ischemia, the hearts were harvested and immediately flash frozen. Five µm sections were incubated with Isolectin B4 (1∶200, IB4, Molecular Probe, Invitrogen) to identify capillaries. The number of capillaries was counted and expressed as capillary density per square millimeter (mm^2^) [12], [16].

### Analysis of VSMC and arteriole formation

To assess VSMC in the border zone of the ischemic area after 24 hours of MI, VSMC were stained with Cy3-conjugated anti-α smooth muscle actin (SMA, 1∶100; Sigma, MO). Arterioles (Ang-1^+^/SMA^+^) was examined at 14 days of MI.

### Myocardial cell/endothelial apoptosis

Heart tissue sections underwent transferase deoxyuridine nick end labeling (TUNEL) following the manufacturer’s instructions (Promega, WI). Sections were counterstained with DAPI. Apoptosis was indexed by counting TUNEL positive cells per square millimeter (mm^2^). To determine endothelial or SMC apoptosis, sections of myocardium were incubated with TUNEL and vWF (1∶200, Santa Cruz, CA) or Cy3-conjugated anti-α smooth muscle actin (SMA, 1∶100; Sigma, MO) overnight, followed by incubation with Fluorolink^TM^ Cy^TM^3 for vWF. Co-localization of TUNEL with vWF or SMA was visualized with a fluorescence microscope (Nikon TE 2000, Japan). Sections were counterstained with DAPI. Endothelial cell/SMC apoptosis was indexed by counting TUNEL/vWF or TUNEL/SMA positive cells per square millimeter (mm^2^).

### Assessment of cardiac function

Briefly, experimental mice were anesthetized with ketamine (100 mg/kg) plus xylazine (15 mg/kg), intubated and artificially ventilated with room air. A 1.4-Fr pressure–conductance catheter (SPR-839, Millar Instrument, Houston, TX) was insert into left ventricle (LV) to record baseline hemodynamics of hearts. Data were digitized with a sampling rate of 1000 Hz. All data were analyzed with LabChart software package from AD instruments.

### Myocardial fibrosis and heart weight to body weight ratio (HW/BW)

To determine cardiac fibrosis formation, 8 µm sections from hearts after 14 days of myocardial ischemia were stained with Masson’s trichrome (MT, Sigma, MO). Myocardial fibrosis was quantified by measuring the MT staining area (blue) in the infarcted area using NIH Image analysis software [4]. Cardiac hypertrophy was assessed by measuring heart-to-body weight ratio at 14 days post-myocardial ischemia. Each heart weight was divided by the total body weight of the mouse, resulting in a ratio representative of cardiac hypertrophy.

### Statistical analysis

All results are expressed as mean ± SD. Statistical analysis was performed using one-way ANOVA followed by the multiple-comparison test (Student–Newman–Keuls or Dunn’s test). A p value <0.05 denoted significance.

## Results

### Overexpression of Ang-1 in diabetic db/db mouse hearts and bone marrow

**Figure 1:**
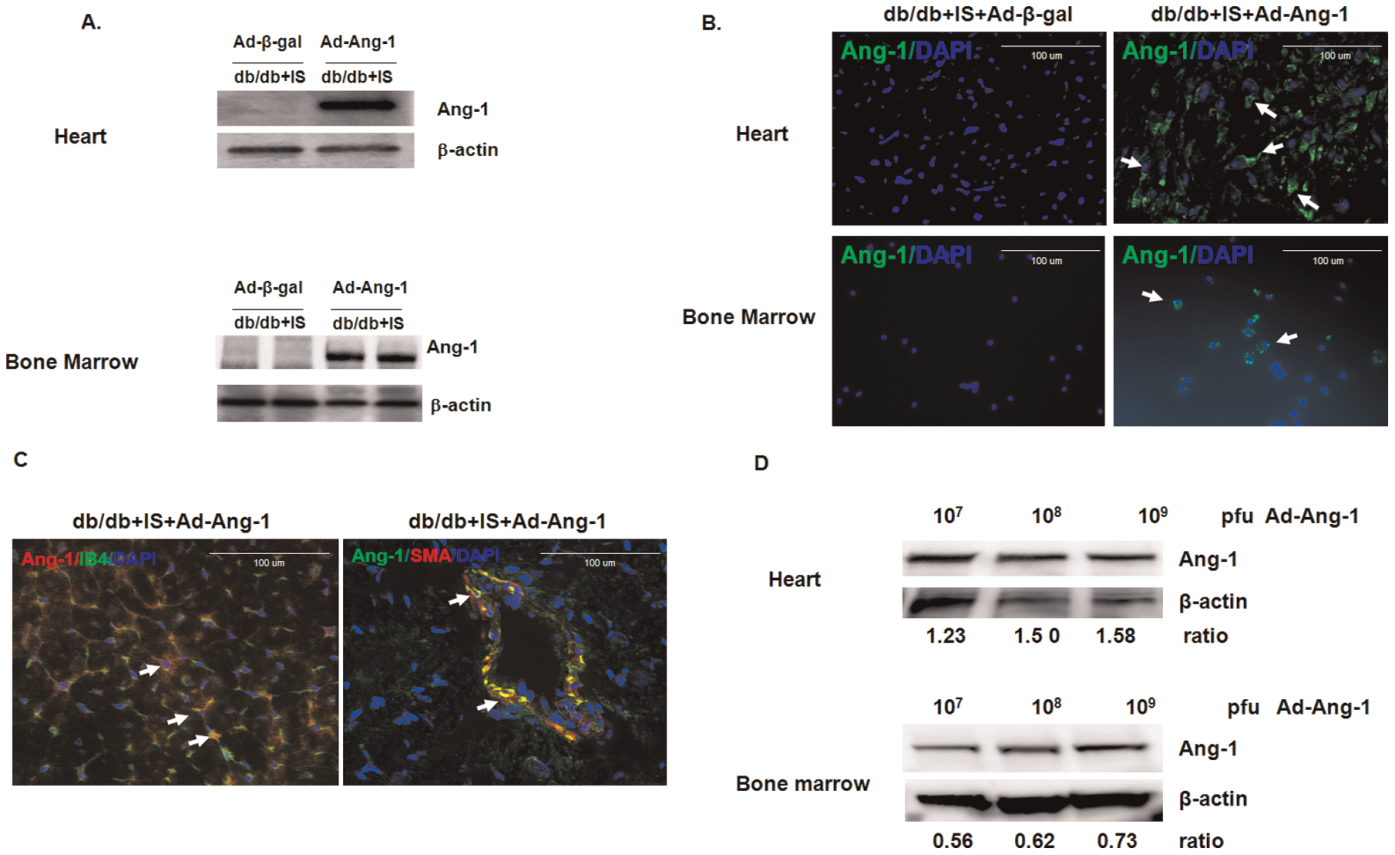
Expression and localization of Ang-1 in the diabetic db/db mouse hearts and bone marrow. (A) Western blot analysis demonstrating that systemic administration of Ad-Ang-1 (1×10^9^ PFU) resulted in overexpression of Ang-1 in the db/db mouse hearts and bone marrow at day 1 compared to mice receiving Ad-β-gal. (B) Ang-1 was stained with an Ang-1 antibody-labeled with FITC (green, 40X) and nuclei were stained with DAPI (blue, 40X). Merged images show Ang-1 expression in Ad-Ang-1 treated db/db mouse hearts and bone marrow (right panel), but not in Ad-β-gal treated db/db mouse hearts and bone marrow (left panel). (C) Ang-1 protein was localized at smooth muscle cell (SMC) and endothelial cell (EC) in heart tissue. Ang-1 expression was imunostained with Texas Red (red). Endothelial cells were stained with LB4 (green). Merged image revealed that Ang-1 was localized to EC (left panel). Ang-1 was stained with Ang-1 linked to FITC (green). SMCs were stained with SMA (Red) and nuclei were stained with DAPI counterstaining (blue). Merged image showed that Ang-1was localized to SMC (right panel). (D) Western blot analysis demonstrating that systemic administration of Ad-Ang-1 (1×10^7^ –1×10^9^ PFU) resulted in overexpression of Ang-1 in the mouse hearts and bone marrow in a dose-dependent manner.

Systemic delivery of Ad-Ang-1 led to dramatic induction of Ang-1 expression in db/db mouse hearts and bone marrow after 24 hours of myocardial ischemia **(** **Figure 1A** **)**. Fluorescent immunohistochemical analysis confirmed the overexpression of Ang-1 in the Ad-Ang-1 treated db/db mouse hearts and bone marrow, but not in the Ad-β-gal treated db/db mouse hearts and bone marrow **(** **Figure 1B** **)**. We also examined the location of Ang-1 expression in Ad-Ang-1–treated *db/db* mouse hearts. Our merged images revealed that Ang-1 localized with Isolectin B4 (IB4) on ECs and SMA on vessel SMCs (**Figure 1C**). To examine transfection efficiency of Ad-Ang-1 *in vivo*, experimental mice were systemic administrated with Ad-Ang-1 at dosage of 10^7^–10^9^ pfu for 24 hours. Western blot analysis showed that Ang-1 protein expression was gradual increased in a dose dependent fashion both in the heart and bone marrow (**Figure 1D**).

### Overexpression of Ang-1 promotes hematopoietic stem cell recruitment via SDF-1α/CXCR-4 signaling in the ischemic hearts of db/db mouse

**Figure 2:**
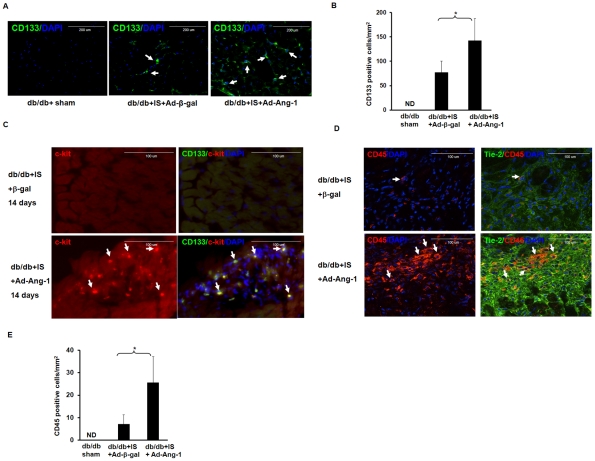
Ang-1 promotes hematopoietic stem cell recruitment into the ischemic hearts of db/db mouse. *A*. Recruitment of CD133^+^ in db/db mouse hearts subjected to myocardial ischemia at 24 hours. The hematopoietic stem cells were stained with a CD133 antibody (green, 40X). The nuclei were stained by DAPI (blue, 40X). *B:* Quantitative analysis of CD133^+^ cells demonstrating that the number of CD133^+^ cells was significantly increased in the Ad-Ang-1 treated db/db mice (n = 6) compared to that of Ad-β-gal-treated db/db mice (n = 9). CD133^+^ cells are expressed as the total number per square mm. Sham control (n = 3). All data represent mean ± SD; *p<0.05. ND =  not detected. *C.* Co-localization of CD133 with c-kit in db/db mouse infarcted hearts at 14 days of MI. CD133^+^ cells (green); c-kit^+^ cells (red) and nuclei were stained by DAPI (blue, 40X). C-kit^+^ cells were recruited into diabetic db/db mouse infarcted hearts in the Ad-Ang-1 treated db/db mice. Merged images further show that CD133^+^ cells were co-localized with c-kit^+^ cells. No c-kit^+^ cells were seen in the Ad-β-gal-treated db/db mice. No specific staining was observed in the sham control group. n = 3 mice each group. *D.* Recruitment of hematopoietic CD45^+^ cells in db/db mouse hearts subjected to myocardial ischemia at 24 hours. The hematopoietic cells were stained with a CD45 antibody (red, 40X). The endothelial cells were stained with a Tie-2 antibody (green). The nuclei were stained by DAPI (blue, 40X). No specific staining was observed in the sham control group. *E:* Quantitative analysis of CD45^+^ cells demonstrating that the number of CD45^+^ cells was significantly increased in the Ad-Ang-1 treated db/db mice (n = 6) compared to that of Ad-β-gal-treated db/db mice (n = 9). CD45^+^ cells are expressed as the total number per square mm. Sham control (n = 3). All data represent mean ± SD; *p<0.05. ND =  not detected.

Next, we investigated whether overexpression of Ang-1 affects recruitment of hematopoietic progenitor cells into infarcted db/db mouse hearts. CD133 and c-kit cells were examined in the ischemic hearts at 24 hour and 14 days. No CD133^+^ cells were observed in the sham control db/db mouse hearts without ischemia. As shown in **Figure 2A and 2B**, CD133^+^ cells were recruited into diabetic mouse infarcted hearts after 24 hours ischemia as compared to Ad-β-gal diabetic mouse infarcted hearts. Overexpression of Ang-1 led to a significant increase in CD133^+^ cells in diabetic infarcted hearts in comparison with Ad-β-gal treated db/db mice. Furthermore, no c-kit^+^ cells were detected in the sham control, db/db+ ischemia and db/db + ischemia+Ad-Ang-1 at 24 hours. Intriguingly, c-kit^+^ cells were detected in the Ad-Ang-1 diabetic mouse infarcted hearts at day 14 of ischemia, but not in Ad-β-gal diabetic mouse. The recruited c-kit^+^ cells were co-localized with CD133^+^ cells in db/db mouse infarcted hearts treated with Ad-Ang-1 (**Figure 2C**).

CD45^+^ positive cells were also dramatically increased in diabetic infarcted hearts treated with Ad-Ang-1 at 24 hours after MI as compared to Ad-β-gal diabetic mouse infarcted hearts. Co-localization studies further revealed that the increased CD45^+^ cells co-localized with Tie-2^+^ in diabetic infarcted hearts treated with Ad-Ang-1 (**Figure 2D and E**).

**Figure 3:**
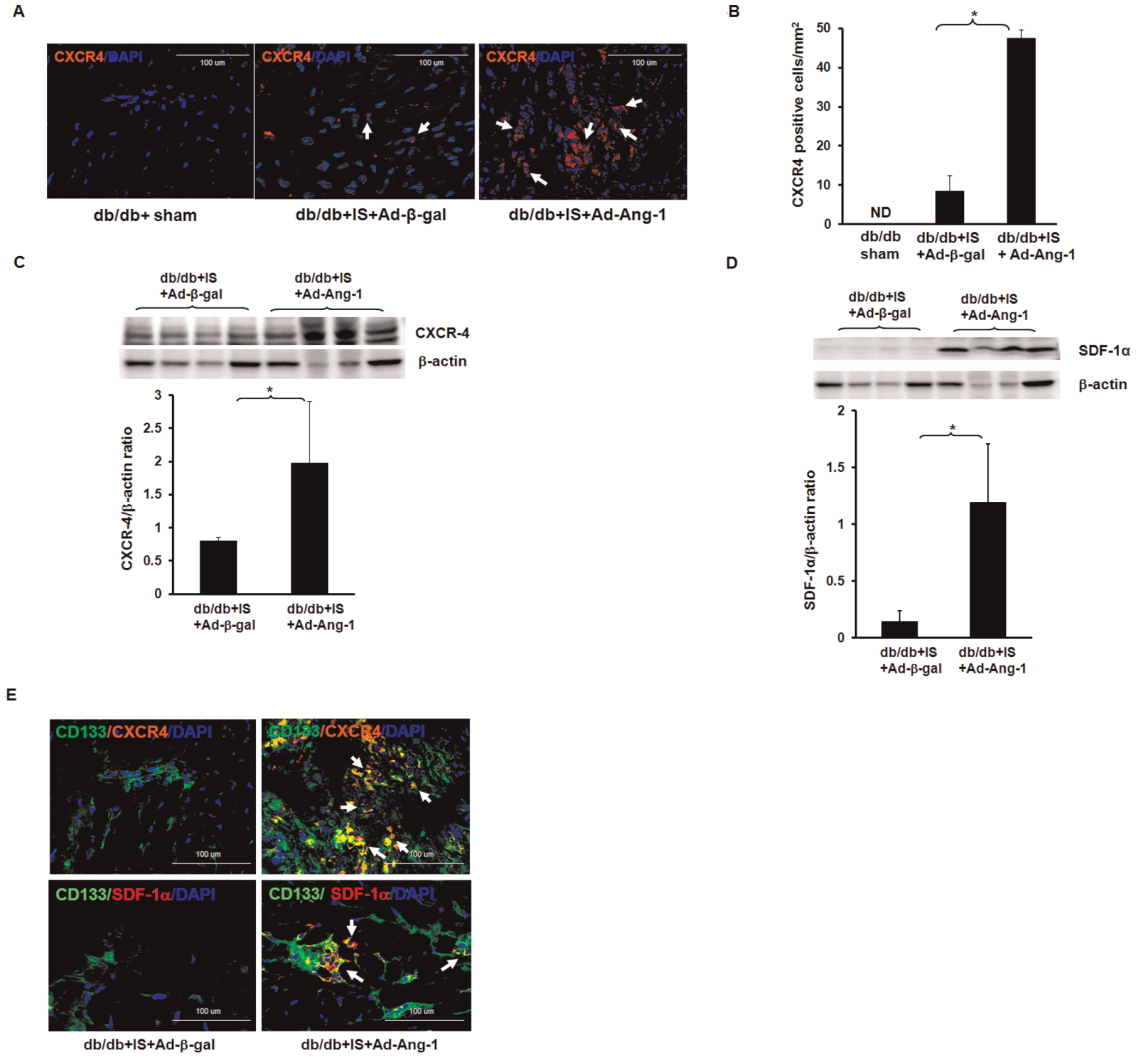
Ang-1 upregulates SDF-1α and CXCR-4 expression in the ischemic hearts of db/db mouse. *A:* Expression of CXCR-4 in db/db mouse hearts subjected to myocardial ischemia at 24 hours. CXCR-4 positive cell (red) and nuclei were stained by DAPI (blue). *B:* Quantitative analysis showing that the number of CXCR-4^+^ cells was significantly increased in the Ad-Ang-1 treated db/db mice (n = 6) compared to that of Ad-β-gal-treated db/db mice (n = 9). CXCR-4^+^ cells are expressed as the total number per square mm. Sham control (n = 3). All data represent mean ± SD; *p<0.05. ND =  not detected. *C:* Western blot and densitometric analysis of myocardial CXCR-4 expression showing that overexpression of Ang-1 significantly increased CXCR-4 expression in db/db mice compared to that of Ad-β-gal-treated db/db mice. All data represent mean ± SD (n = 4 mice); *p<0.05. *D.* Western blot and densitometric analysis of myocardial SDF-1α expression. Overexpression of Ang-1 significantly increased SDF-1α expression in db/db mice compared to that of Ad-β-gal-treated db/db mice. All data represent mean ± SD (n = 4 mice); *p<0.05. *E.* Co-localization of CD133 with CXCR-4 and CD133 with SDF-1α in db/db mouse infarcted hearts. CD133^+^ cells (green); CXCR-4^+^ or SDF-1α^+^ cells (red) and nuclei were stained by DAPI (blue, 40X). Merged images show that CD133^+^ cells co-localized with both CXCR-4 (upper panel) and SDF-1α (bottom panel) in the Ad-Ang-1 treated db/db mice, but little co-localization was seen in the Ad-β-gal-treated db/db mice. No specific staining was observed in the sham control group.

To examine the roles of SDF-1α/CXCR-4 in Ang-1-induced CD133^+^ cell recruitment into db/db mouse hearts, we examined the number of CXCR-4^+^ cells and SDF-1α/CXCR-4 protein expression in the border zone of infarcted myocardium after 24 hours of MI. As shown in **Figure 3A and B**, overexpression of Ang-1 in db/db mice led to a significant increase in the number of CXCR-4^+^ cells in the border zone of infarcted hearts in comparison with Ad-β-gal treated db/db mice**.** No CXCR-4^+^ cells were found in the sham control db/db mouse hearts. Western blot analysis revealed that overexpression of Ang-1 resulted in significant increases in CXCR-4 and SDF-1α expression in diabetic infarcted hearts as compared to Ad-β-gal diabetic mouse infarcted hearts **(** **Figure 3** **C and D)**. Co-localization studies further revealed that the increased CD133^+^ co-localized with CXCR-4^+^ and SDF-1α^ +^ cells in diabetic infarcted hearts treated with Ad-Ang-1 **(** **Figure 3E** **)**.

### Overexpression of Ang-1 attenuates myocardial apoptosis and reduces cardiac fibrosis in diabetic db/db mouse hearts

**Figure 4:**
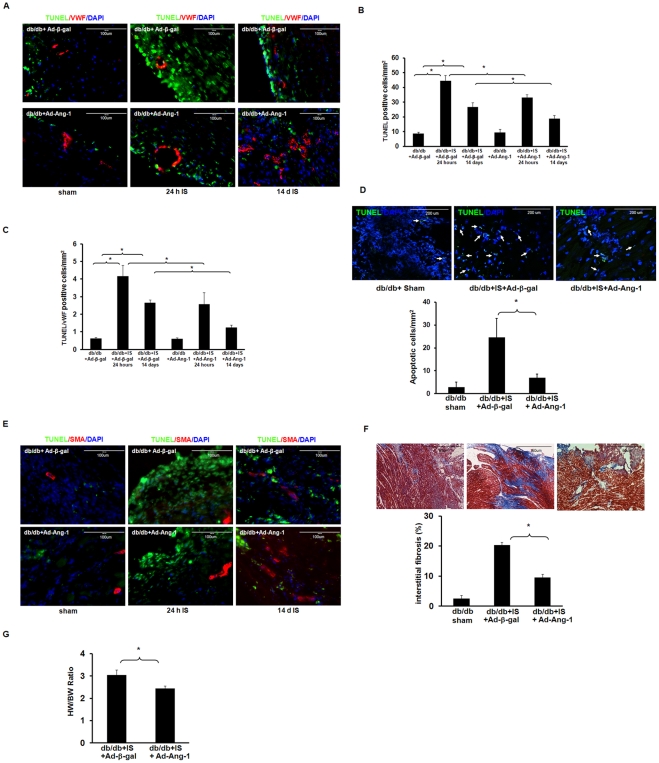
Ang-1 attenuates myocardial/endothelial apoptosis, and reduces cardiac fibrosis and hypertrophy in db/db mouse. *A.* TUNEL-stained heart sections from sham control db/db mouse, db/db mouse treated with Ad-β-gal or Ad-Ang-1 at 24 hours and 14 days of MI. Myocardial and endothelial apoptotic cells in the infarcted area of the ischemic hearts were identified by TUNEL staining (green) and TUNEL/vWF (red) positive staining, and total nuclei by DAPI counterstaining (blue). *B and C.* Quantitative analysis of apoptotic cells in sham control db/db mouse and infarcted area of db/db mouse treated with Ad-β-gal or Ad-Ang-1 at 24 hours and 14 days after ischemia. TUNEL or TUNEL/vWF positive cells are expressed as the total number of per mm^2^. Apoptotic cells were significantly decreased in Ad-Ang-1 compared to Ad-β-gal mice. All data represent mean ± SD (n = 4 mice); *p<0.05. *D.* Representative images and quantitative analysis of apoptotic cells in the remote area of infarction of db/db mouse at 24 hours after ischemia. Apoptotic cells were significantly decreased in Ad-Ang-1 treated db/db mouse hearts (n = 5) compared to Ad-β-gal treated db/db mice (n = 7). All data represent mean ± SD; sham control n = 5, *p<0.05. *E.* Representative images of TUNEL and SMA-stained heart sections from sham control db/db mouse, db/db mouse treated with Ad-β-gal or Ad-Ang-1 at 24 hours and 14 days of MI. Myocardial and SMCs apoptotic cells in the infarcted area of the ischemic hearts were identified by TUNEL (green) and SMA (red) positive staining, and total nuclei by DAPI counterstaining (blue). *F.* Representative images of cardiac fibrosis formation and quantitative analysis of cardiac fibrosis area in db/db mice treated with Ad-β-gal or Ad-Ang-1 stained by Masson’s trichrome. Ad-Ang-1 significantly attenuated area of cardiac fibrosis in db/db mice compared to Ad-β-gal mice. All data represent mean ± SD (n = 5 mice); *p<0.05. *G.* Heart weight/body weight (HW/BW) ratio in db/db and Ad-Ang-1-treated db/db mouse hearts at 14 days after MI. Treatment with Ad-Ang-1 significantly attenuated cardiac hypertrophy in db/db mouse hearts (n = 5) compared to Ad-β-gal mice (n = 4). All data represent mean ± SD; *p<0.05.

We further sought to determine whether increased recruitment of CD133^+^/c-kit^+^ cells into the ischemic area by Ang-1 would minimize myocardial apoptosis. TUNEL staining and quantitative analysis revealed a significant increase in TUNEL-positive cells in the infarcted area of db/db mice after 24 hours and 14 days of MI compared to sham control db/db mice. db/db mice treated with Ad-Ang-1 had significantly fewer TUNEL-positive cells in the infarct area compared to db/db mice that received Ad-β-gal **(** **Figure 4A and B** **)**. Co-staining of TUNEL with vWF further revealed that treatment of db/db mice with Ad-Ang-1 significantly attenuated the number of TUNEL/vWF-positive cells in the infarct area compared to db/db mice that received Ad-β-gal **(** **Figure 4A and C** **)**. Similarly, treatment of db/db mice with Ad-Ang-1 for 24 hours significantly reduced myocardial apoptosis in the remote area of ischemia compared to db/db mice that received Ad-β-gal **(** **Figure 4D** **)**. However, treatment with Ad-Ang-1 has little effect on the number of TUNEL/SMA positive cells **(** **Figure 4E** **).** Compared to db/db mice treated with Ad-β-gal, mice treated with Ad-Ang-1 showed a significant decrease in the area of myocardial fibrosis at 14 days after MI **(** **Figure 4F** **)**. At 14 days, treatment with Ad-Ang-1 led to a significant decrease in the HW/BW ratio in db/db mice subjected to MI as compared with db/db mice treated with Ad-β-gal (**Figure 4G**).

### Overexpression of Ang-1 increases the number of BM-CD31^+^ cells and myocardial capillary density in db/db mouse hearts

**Figure 5:**
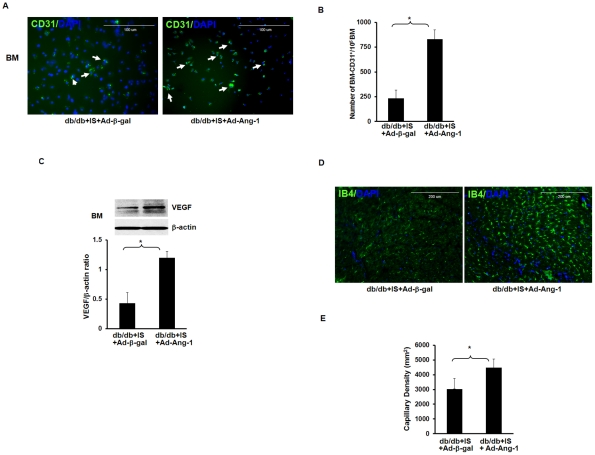
Ang-1 increases CD31^+^ cells and VEGF expression in the BM and promotes capillary formation in db/db mouse hearts. *A.* Representative images showing that BM cells differentiate into CD31^+^ cells in Ad-β-gal treated db/db mice subjected to myocardial ischemia for 24 hours (left panel) and in the Ad-Ang-1treated db/db mice (right panel). *B.* Quantitative analysis of BM differentiation into CD31^+^ after myocardial ischemia. The number of BM cells that differentiate into CD31^+^ was significantly increased in the Ad-Ang-1treated db/db mice compared to the Ad-β-gal treated db/db mice. There was not difference between sham control and ischemic group. All data represent mean ± SD (n = 3 mice); *p<0.05. *C.* Western blot densitometric analysis of VEGF expression revealed that systemic delivery of Ad-Ang-1 resulted in a significant increase in VEGF expression in the BM of db/db mouse compared to the Ad-β-gal db/db treated mice. All data represent mean ± SD (n = 3 mice); *p<0.05. *D and E.* Representative images and quantitative analysis showing that treatment with Ad-Ang-1 significantly increased capillary formation in db/db mice subjected to ischemia compared to Ad-â-gal-treated db/db mice. All data represent mean ± SD (n = 7 mice); *p<0.05.

New evidence indicates that BM-CD31+ cells have angiogenic properties and implantation of BM-CD31^+^ cells into ischemic hind-limb promotes recovery of tissue from ischemic injury [17]. Therefore, we examined the number of BM-CD31^+^ cells after 24 hours of myocardial ischemia. The number of BM-CD31^+^ was significantly increased in db/db mice treated with Ad-Ang-1 compared to db/db mice treated with Ad-β-gal (**Figure 5A and B**). Overexpression of Ang-1 in BM also resulted in a significant increase in VEGF expression (**Figure 5C**). Further, overexpression of Ang-1 in db/db mouse hearts resulted in a significant increase in myocardial capillary density at 14 days in the border zone of infarcted myocardium compared to db/db mice treated with Ad-β-gal **(** **Figure 5D and E** **)**. The sham control had little effect on BM-CD31^+^ cells and myocardial capillary density (data not shown).

### Overexpression of Ang-1 increases BM-SMA^+^ cells and number of VSMC in the db/db mouse hearts

**Figure 6:**
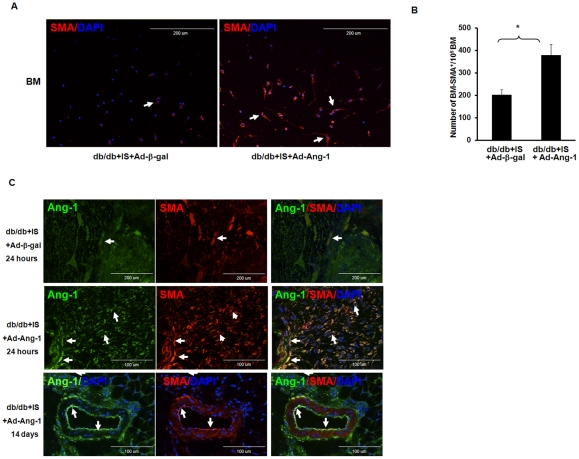
Ang-1 increases number of SMA^+^ cells in the db/db mouse BM and hearts. *A.* Representative images showing that BM cells differentiate into vascular smooth muscle-like (SMA^+^) cells in Ad-β-gal treated db/db mice subjected to MI for 24 hours (left panel) and in the Ad-Ang-1treated db/db mice (right panel). *B.* Quantitative analysis showing a significant increase in number of SMA^+^ cells after myocardial ischemia for 24 hours following Ad-Ang-1 compared to Ad-β-gal in db/db mice subjected to MI. All data represent mean ± SD (n = 3 mice); *p<0.05. *C.* Representative images of myocardial VSMC in infarcted areas in db/db+IS+Ad-β-gal and db/db+IS+Ad-Ang-l mice stained by Ang-1^+^/SMA^+^ cells. Ang-1 was stained with monoclonal anti-angiopoietin-1 linked to FITC (green, 40X). VSMCs were stained with smooth muscle actin (SMA, Red, 40X) and nuclei were stained with DAPI counterstaining (blue, 40x). Merged image showed that over-expressed Ang-1 co-localized with VSMC. Top and middle panel: representative images showing that systemic delivery of Ad-Ang-1 led to a dramatic increase in the number of Ang-1^+^/SMA^+^ cells in db/db mouse subjected to myocardial ischemia for 24 hours compared to Ad-β-gal in db/db mice. Bottom panel: representative images showing the newly formed arteriole (Ang-1^+^/ SMA^+^) in the Ad-Ang-1 treated db/db mouse heart after ischemia for 14 days.

The number of BM-SMA^+^ cells was significantly increased in db/db mice treated with Ad-Ang-1 compared to db/db mice treated with Ad-β-gal after MI (**Figure 6A & B**). Next, the number of VSMC in the ischemic area was examined after 24 hours of ischemia by the co-localization of Ang-1 with SMA. Ang-1 expression co-localized with SMA (middle panel). After 2 weeks of MI, Ang-1-positive arteriole with large diameter was also found in the border zone of infarcted myocardial area **(** **Figure 6C** **)**. Sham control had little effect on BM-SMA^+^ and VSMC cell number (Data not shown).

### Overexpression of Ang-1 upregulates Jagged1/Notch3 and apelin expression in diabetic db/db mouse hearts

**Figure 7:**
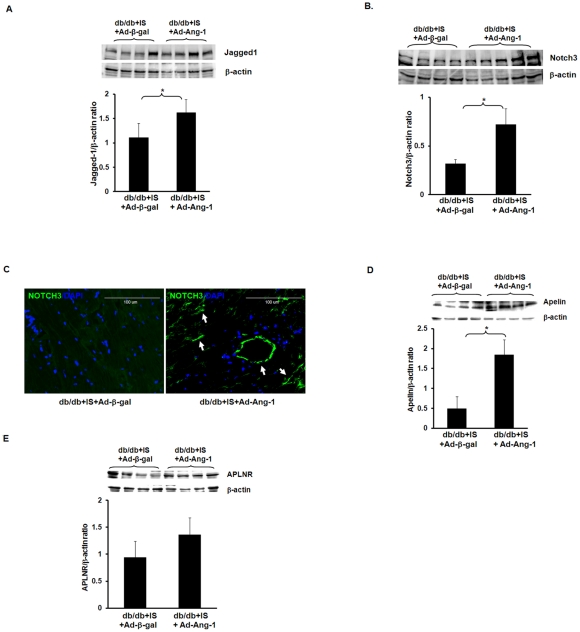
Ang-1 upregulates Jagged1, Notch3 and apelin expression in db/db mouse hearts. *A.* Western blot densitometry analysis of myocardial Jagged 1 expression showing that systemic delivery of Ad-Ang-1 resulted in a significant increase in Jagged 1 expression in the db/db mice subjected to myocardial ischemia for 24 hours compare to the db/db mice treated with Ad-β-gal. All data represent mean ± SD (n = 4 mice); *p<0.05. *B.* Western blot densitometric analysis of myocardial Notch3 expression revealing that systemic delivery of Ad-Ang-1 (n = 5) resulted in a significant increase in Notch3 expression in db/db mice subjected to myocardial ischemia for 24 hours compare to the db/db mice treated with Ad-β-gal (n = 4). All data represent mean ± SD; *p<0.05. *C.* Immunofluorescence microscopy showing expression of Notch3 in the vascular wall of db/db mice infarcted hearts treated with Ad-Ang-1. Merged images showed that Notch3 expression was expressed in newly formed arteriole of the Ad-Ang-1 treated db/db mice (right panel), but not the Ad-β-gal-treated db/db mice (left panel). Notch3 was stained with Notch antibody conjugated to FITC (green, 40X) and nuclei were stained by DAPI (blue). No specific staining was observed in the sham control group. *D.* Western blot densitometry analysis showing that apelin expression was significantly increased in the db/db mice treated with Ad-Ang-1 compare to the Ad-β-gal treated db/db mice at 24 hours of ischemia. All data represent mean ± SD (n = 4 mice); *p<0.05. *E.* APLNR expression was increased in the Ad-Ang-1 treated db/db mice compare to the Ad-β-gal treated db/db mice at 24 hours of ischemia, but did not achieve a statistical significantly difference. All data represent mean ± SD (n = 4 mice); p>0.05.

To explore the potential intracellular molecular mechanisms by which overexpression of Ang-1 increased angiogenesis in diabetes, myocardial Jagged 1/Notch3 and apelin/APLNR expression in db/db mice was examined. Systemic delivery of Ad-Ang-1 resulted in a significant increase in Jagged 1 and Notch3 expression 24 hours after myocardial ischemia compared to Ad-β-gal **(** **Figure 7A and B** **)**. Immunohistochemical analysis confirmed that the increased Notch3 localized in the wall of vessels in diabetic infarcted hearts treated with Ad-Ang-1 (**Figure 7C**). Systemic delivery of Ad-Ang-1 also leads to a significant increase in apelin expression at 24 hours after MI compared to Ad-β-gal **(** **Figure 7D** **)**. APLNR (APJ) expression in the db/db mice treated with Ad-Ang-1 showed a trend to increase, however, failed to achieve statistical significance **(** **Figure 7E** **)**. Sham control had little effect on Jagged1/Notch3 and apelin/APLNR expression (Data not shown).

### Overexpession of Ang-1 improves diabetic cardiac function after myocardial ischemia

**Figure 8:**
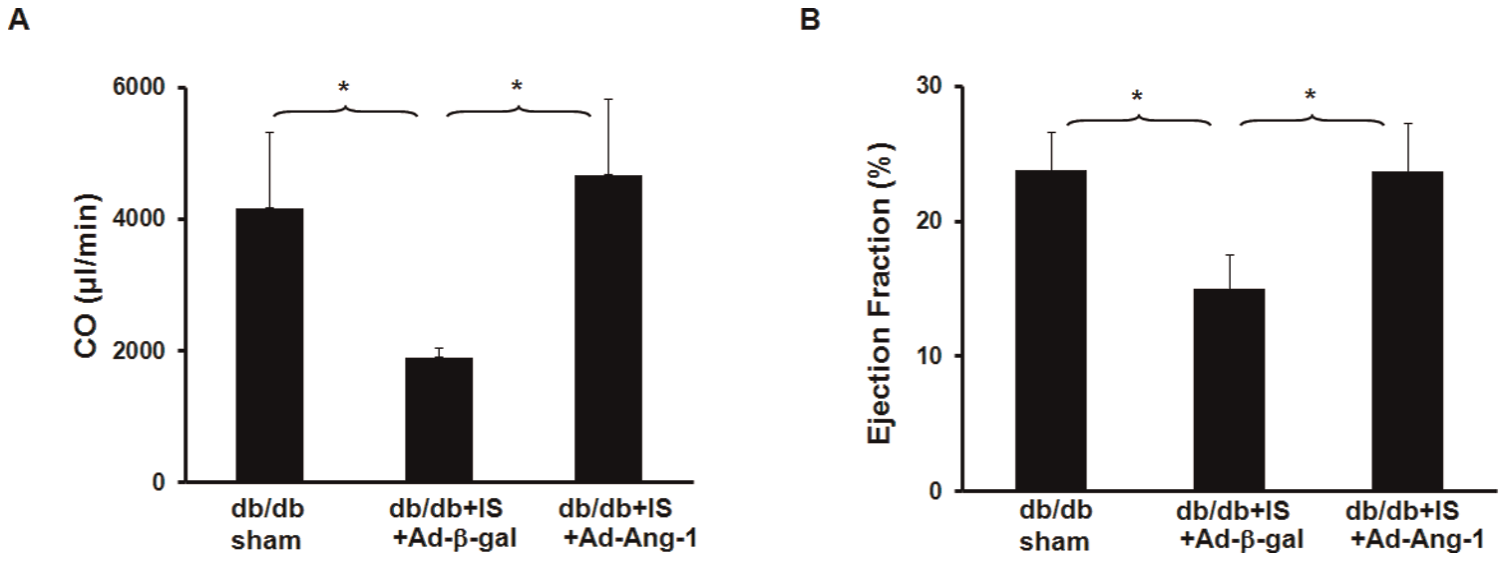
Overexpression of Ang-1 improves cardiac functional recovery of post-MI in db/db mice. *A.* Cardiac output (CO) was significantly improved in the db/db mice treated with Ad-Ang-1 (n = 5) compare to the Ad-β-gal treated db/db mice (n = 4) at 14 days of ischemia. Sham control (n = 4). All data represent mean ± SD; *p<0.05. *B.* Myocardial ejection fraction (EF%) was significantly increased in the db/db mice treated with Ad-Ang-1 (n = 5) compare to the Ad-β-gal treated db/db mice (n = 4) at 14 days of ischemia. Sham control (n = 4). All data represent mean ± SD; *p<0.05.

To determine whether increased homing of hematopoietic stem cell and angiogenesis by Ang-1 promoted cardiac functional recovery, cardiac function was measured by P-V loop at 14 days of myocardial ischemia. As shown in Fig 8 A and B, exposure of diabetic hearts to ischemia for 14 days resulted in a dramatic reduction of cardiac output (CO) and ejection fraction (EF%). Overexpression of Ang-1 led to a significant improvement of CO and EF% in diabetic infarcted hearts.

## Discussion

Our data establish, for the first time, that Ang-1 plays a critical role in the recruitment of hematopoietic stem cell CD133^+^/c-kit^+^ and CD45^+^ cells into ischemic areas of the diabetic infarcted heart. This is accompanied with a significant reduction in myocardial/endothelial apoptosis and cardiac hypertrophy as well as fibrosis formation in diabetic heart. Our study also demonstrates that upregulation of SDF-1α/CXCR-4 expression in the diabetic infarcted hearts is a novel downstream signaling pathway of Ang-1-mediated recruitment of CD133^+^ cells. Further, our studies reveal that overexpression of Ang-1 results in increases in capillary density and arteriole formation together with a dramatic improvement of cardiac functional recovery in the diabetic infarcted hearts. These studies strongly suggest that Ang-1 gene therapy protects the diabetic heart from ischemic injury by promoting CD133^+^ and c-kit^+^ stem cell recruitment, and enhancing angiogenesis via a mechanism involving SDF-1α/CXCR-4 signaling.

Increasing evidence reveals that Ang-1 has a burgeoning role in heart disease and possesses cardioprotective effects against myocardial ischemia [18] ^,^ [19]. Previously, we showed that overexpression of Ang-1 increases myocardial angiogenesis and decreases myocardial infarction in STZ mouse model, indicating a potential link between angiogenesis and reduction of MI in diabetes [12]. The present studies further reveal that overexpression of Ang-1 in db/db mouse hearts results in a significant reduction in myocardial apoptosis as well as endothelial cell apoptosis. This was accompanied by significant increase in capillary density and cardiac functional recovery. Although overexpression of Ang-1 alleviates myocardial ischemic injury in the STZ and db/db diabetic animal models, [4], [12] the underlying mechanisms by which overexpression of Ang-1 attenuates myocardial apoptosis and promotes cardiac repair are not completely understood.

Previous studies showed that Tie-2 expression was detected in hematopoietic stem cell and Tie-2 signaling was crucial for the maintenance of the hematopoietic microenvironment. Ang-1 actives Tie-2 on hematopoietic stem cell in the niche, maintains the *in vivo* repopulation ability of hematopoietic stem cell.[13] Recent evidence suggests that hematopoietic stem cell CD133^+^ cells are involved in healing the ischemic myocardium and thus may be important in functional recovery of the myocardium following acute infarction (AMI) [20]–[22]. In non-diabetic patients with AMI, there is often a surge of CD133^+^ cells in the circulation. However, in diabetic patients experiencing AMI, CD133^+^ cell recruitment and function was significantly impaired [20]. CD133^+^ cells have been reported to release large amounts of VEGF, which activates PI3K/Akt and exerts pro-angiogenic and pro-survival effects. Transplantation of a low number of CD133^+^ human fetal aorta-derived vascular progenitor cells promotes reparative neovascularization and skeletal myocyte regeneration in a non-diabetic ischemic hind-limb model [22]. Intriguingly, local therapy with CD133^+^ cells promotes angiogenesis and significantly accelerates healing of ischemic hind-limb skin wounds in a diabetes mellitus model [21]. To our knowledge, the present study is the first demonstration showing that overexpression of Ang-1 significantly increases the recruitment of CD133^+^ and c-kit^+^ into myocardial ischemic areas in diabetic db/db mice. Previously, we have shown that overexpression of Ang-1 significantly increases myocardial VEGF, Akt and eNOS expression in db/db mice [4]. Based upon these findings, we postulate that Ang-1 promotes CD133^+^ cell and c-kit^+^ recruitment into ischemic areas, leading to secretion of VEGF and activation of the Akt/eNOS signaling pathway, which exert beneficial effects on the diabetic infarcted hearts. This notion is supported by our findings that CXCR-4 and SDF-1α, the chemokine for hematopoietic progenitor cells, were significantly increased within the ischemic myocardium of Ang-1 treated db/db mouse. Furthermore, our data reveal that the increased CD133^+^ co-localize with CXCR-4^+^ and SDF-1α^ +^ cells in the area of myocardial ischemia. In addition, SDF-1α/CXCR-4 has been shown to protect the hearts after myocardial infarction [14], [23], [24]. These findings suggest that activation of SDF-1α/CXCR-4 signaling mediates Ang-1-induced recruitment of CD133^+^ progenitor cells into the infarcted ischemic heart leading to attenuation of myocardial apoptosis. In addition, our present study shows that Ang-1 protects myocardial endothelial cell against ischemia-induced endothelial apoptosis. Our previous study showed that Ang-1 increases myocardial angiogenesis via activation of Tie-2/Akt/eNOS pathway in coronary endothelial cells [25]. These direct protective effects of Ang-1 on endothelial cells may also contribute to the attenuation of myocardial ischemic injury and increase in myocardial angiogenesis and cardiac function in diabetes.

Ang-1/Tie-2 signaling plays a predominant role in controlling both VSMC and pericyte maturation and is essential for the maintenance of vascular stabilization [1], [26]. We have demonstrated that overexpression of Ang-1 increases VSMC recruitment and promotes mature neovessel formation; this is accompanied by a significant increase in capillary density in db/db mouse hearts [4]. However, the intracellular molecular mechanisms by which Ang-1 increases VSMC recruitment and maturation remains unexplored. The Notch ligand Jagged1 is essential for vascular remodeling and has been linked to congenital heart failure in humans [27]. Jagged1 plays a critical role in the regulation of VSMC recruitment/maturation via Notch3 during early embryonic development [28]. Deficiency of Notch3 has been shown to disrupt VSMC differentiation and to increase infarct size in ischemic stroke [29]–[31]. Recent studies reveal that ethanol and cyclic strain stimulate endothelial cell angiogenic activity via a Notch/Ang-1/Tie-2 pathway suggesting potential cross-talk between Notch signaling and the Ang-1/Tie-2 pathway [32], [33]. The present data show that overexpression of Ang-1 significantly increased Jagged 1 and Notch3 expression in the db/db mouse hearts. Immunohistochemical analysis further confirmed that the Notch3 expression was localized to the wall of larger arterioles. Apelin is an endogenous ligand of the human orphan G-protein-coupled receptor APJ [34], [35]. Apelin exerts a variety of cardiovascular effects and particularly acts as an activator of angiogenesis [36]–[39]. A recent study also reported that the apelin/APJ system was involved in the regulation of blood vessel diameter during angiogenesis [40]. Our present data also demonstrated that treatment with Ad-Ang-1 significantly increase apelin expression. These data suggest potential role of Jagged1/Notch3 and apelin in Ang-1-mediated angiogenesis. Most intriguingly, overexpression of Ang-1 increases the number of BM-SMA^+^ cells as well as Ang-1^+^/SMA^+^ cells in the diabetic infarcted hearts, suggesting that Ang-1 over-expressing SMA^+^ cells in the BM may also be recruited to the ischemic myocardium rather than their homing to Ang-1^+^ cells in the heart itself.

Recruited CD45^+^ cells represent a rich source of hematopoietic stem cell cytokines in the ischemic heart.[41] Our data showed that CD45^+^ cells were dramatically increased in diabetic infarcted hearts after Ad-Ang-1 treatment. Furthermore, the abundance Tie-2-expressing CD45^+^ cell was increased implying that these cells may contribute to the Ang-1-mediated angiogenic response in diabetic infarcted hearts. Our data also demonstrate that overexpression of Ang-1 significantly increased BM-derived CD31^+^ cells and upregulated VEGF expression in BM. Recent studies showed that BM-CD31^+^ cells were enriched with remarkably higher levels of angiogenic and hematopoietic genes as compared to BM-CD31^−^ cells. Furthermore, BM-CD31^+^ cells induced the activation of angiogenic, anti-apoptotic and chemo-attractant factors in ischemic hind-limbs via a paracrine effect. Transplantation with BM-CD31^+^ cells significantly increased angiogenesis and improved recovery from ischemia 42,43. Jagged1 has been shown to drive immature BM progenitor cells to differentiate into functional EPCs [44]. Jagged1 and Notch3 were increased during the differentiation of BM-derived mononuclear cells into VSMC and EC [45]. Further studies are needed to elucidate the molecular mechanisms and crosstalk between the Jagged1/Notch3 and Ang-1/Tie-2 pathways in the regulation of BM/stem cell recruitment/differentiation into VSMC/EC and vascular maturation.

The present study demonstrates that following myocardial ischemia, Ang-1 gene therapy promotes hematopoietic stem cell recruitment. These changes are associated with an increase in CXCR-4/SDF-1α expression and myocardial angiogenesis and with a reduction in cardiac hypertrophy and improvement of cardiac functional recovery after ischemia. Given the roles of Ang-1/Tie-2 signaling in hematopoietic stem cell development as well as EC/VSMCs differentiation, our data provide novel insights into the intracellular pathways that regulate the formation of mature vasculature in the diabetic infarcted heart and mechanisms that promote cardiac repair. Our studies may aid in the development of novel therapeutic avenues to ameliorate the impaired stem cell function and insufficient angiogenesis that are characteristic of the diabetic state.

## References

[1] Suri C, Jones PF, Patan S, Bartunkova S, Maisonpierre PC (1996). Requisite role of angiopoietin-1, a ligand for the TIE2 receptor, during embryonic angiogenesis.. Cell.

[2] Papapetropoulos A, Fulton D, Mahboubi K, Kalb RG, O’Connor DS (2000). Angiopoietin-1 inhibits endothelial cell apoptosis via the Akt/survivin pathway.. J Biol Chem.

[3] Joussen AM, Poulaki V, Tsujikawa A, Qin W, Qaum T (2002). Suppression of diabetic retinopathy with angiopoietin-1.. Am J Pathol.

[4] Chen JX, Stinnett A (2008). Ang-1 gene therapy inhibits hypoxia-inducible factor-1alpha (HIF-1alpha)-prolyl-4-hydroxylase-2, stabilizes HIF-1alpha expression, and normalizes immature vasculature in db/db mice.. Diabetes.

[5] Isner JM, Asahara T (1999). Angiogenesis and vasculogenesis as therapeutic strategies for postnatal neovascularization.. J Clin Invest.

[6] Schachinger V, Assmus B, Britten MB, Honold J, Lehmann R (2004). Transplantation of progenitor cells and regeneration enhancement in acute myocardial infarction: final one-year results of the TOPCARE-AMI Trial.. J Am Coll Cardiol.

[7] Britten MB, Abolmaali ND, Assmus B, Lehmann R, Honold J (2003). Infarct remodeling after intracoronary progenitor cell treatment in patients with acute myocardial infarction (TOPCARE-AMI): mechanistic insights from serial contrast-enhanced magnetic resonance imaging.. Circulation.

[8] Hu CH, Li ZM, Du ZM, Zhang AX, Rana JS (2010). Expanded human cord blood-derived endothelial progenitor cells salvage infarcted myocardium in rats with acute myocardial infarction.. Clin Exp Pharmacol Physiol.

[9] Glass CE, Singal PK, Singla DK (2010). Stem cells in the diabetic infarcted heart..

[10] Thum T, Fraccarollo D, Schultheiss M, Froese S, Galuppo P (2007). Endothelial nitric oxide synthase uncoupling impairs endothelial progenitor cell mobilization and function in diabetes.. Diabetes.

[11] Yoon YS, Uchida S, Masuo O, Cejna M, Park JS (2005). Progressive attenuation of myocardial vascular endothelial growth factor expression is a seminal event in diabetic cardiomyopathy: restoration of microvascular homeostasis and recovery of cardiac function in diabetic cardiomyopathy after replenishment of local vascular endothelial growth factor.. Circulation.

[12] Tuo QH, Zeng H, Stinnett A, Yu H, Aschner JL (2008). Critical role of angiopoietins/Tie-2 in hyperglycemic exacerbation of myocardial infarction and impaired angiogenesis.. Am J Physiol Heart Circ Physiol.

[13] Arai F, Hirao A, Ohmura M, Sato H, Matsuoka S (2004). Tie2/angiopoietin-1 signaling regulates hematopoietic stem cell quiescence in the bone marrow niche..

[14] Askari AT, Unzek S, Popovic ZB, Goldman CK, Forudi F (2003). Effect of stromal-cell-derived factor 1 on stem-cell homing and tissue regeneration in ischaemic cardiomyopathy..

[15] Chen JX, Zeng H, Tuo QH, Yu H, Meyrick B (2007). NADPH oxidase modulates myocardial Akt, ERK1/2 activation and angiogenesis after hypoxia/reoxygenation.. Am J Physiol Heart Circ Physiol.

[16] Chen JX, Stinnett A (2008). Disruption of Ang-1/Tie-2 signaling contributes to the impaired myocardial vascular maturation and angiogenesis in type II diabetic mice.. Arterioscler Thromb Vasc Biol.

[17] Kim H, Cho HJ, Kim SW, Liu B, Choi YJ (2010). CD31+ cells represent highly angiogenic and vasculogenic cells in bone marrow: novel role of nonendothelial CD31+ cells in neovascularization and their therapeutic effects on ischemic vascular disease..

[18] Dallabrida SM, Ismail NS, Pravda EA, Parodi EM, Dickie R (2008). Integrin binding angiopoietin-1 monomers reduce cardiac hypertrophy..

[19] Takahashi K, Ito Y, Morikawa M, Kobune M, Huang J (2003). Adenoviral-delivered angiopoietin-1 reduces the infarction and attenuates the progression of cardiac dysfunction in the rat model of acute myocardial infarction..

[20] Voo S, Dunaeva M, Eggermann J, Stadler N, Waltenberger J (2009). Diabetes mellitus impairs CD133+ progenitor cell function after myocardial infarction..

[21] Barcelos LS, Duplaa C, Krankel N, Graiani G, Invernici G (2009). Human CD133+ progenitor cells promote the healing of diabetic ischemic ulcers by paracrine stimulation of angiogenesis and activation of Wnt signaling..

[22] Invernici G, Madeddu P, Emanueli C, Parati EA, Alessandri G (2008). Human fetal aorta-derived vascular progenitor cells: identification and potential application in ischemic diseases..

[23] Hu X, Dai S, Wu WJ, Tan W, Zhu X (2007). Stromal cell derived factor-1 alpha confers protection against myocardial ischemia/reperfusion injury: role of the cardiac stromal cell derived factor-1 alpha CXCR4 axis..

[24] Frederick JR, Fitzpatrick JR, III, McCormick RC, Harris DA, Kim AY (2010). Stromal cell-derived factor-1alpha activation of tissue-engineered endothelial progenitor cell matrix enhances ventricular function after myocardial infarction by inducing neovasculogenesis..

[25] Chen JX, Lawrence ML, Cunningham G, Christman BW, Meyrick B (2004). HSP90 and Akt modulate Ang-1-induced angiogenesis via NO in coronary artery endothelium.. J Appl Physiol.

[26] Thurston G, Rudge JS, Ioffe E, Zhou H, Ross L (2000). Angiopoietin-1 protects the adult vasculature against plasma leakage.. Nat Med.

[27] Suchting S, Freitas C, le NF, Benedito R, Breant C (2007). Negative regulators of vessel patterning.. Novartis Found Symp.

[28] High FA, Jain R, Stoller JZ, Antonucci NB, Lu MM (2009). Murine Jagged1/Notch signaling in the second heart field orchestrates Fgf8 expression and tissue-tissue interactions during outflow tract development.. J Clin Invest.

[29] Dichgans M (2007). Genetics of ischaemic stroke.. Lancet Neurol.

[30] Joutel A, Monet-Lepretre M, Gosele C, Baron-Menguy C, Hammes A (2010). Cerebrovascular dysfunction and microcirculation rarefaction precede white matter lesions in a mouse genetic model of cerebral ischemic small vessel disease.. J Clin Invest.

[31] rboleda-Velasquez JF, Zhou Z, Shin HK, Louvi A, Kim HH (2008). Linking Notch signaling to ischemic stroke.. Proc Natl Acad Sci U S A.

[32] Morrow D, Cullen JP, Cahill PA, Redmond EM (2007). Cyclic strain regulates the Notch/CBF-1 signaling pathway in endothelial cells: role in angiogenic activity.. Arterioscler Thromb Vasc Biol.

[33] Morrow D, Cullen JP, Cahill PA, Redmond EM (2008). Ethanol stimulates endothelial cell angiogenic activity via a Notch- and angiopoietin-1-dependent pathway.. Cardiovasc Res.

[34] O’Dowd BF, Heiber M, Chan A, Heng HH, Tsui LC (1993). A human gene that shows identity with the gene encoding the angiotensin receptor is located on chromosome 11.. Gene.

[35] Tatemoto K, Hosoya M, Habata Y, Fujii R, Kakegawa T (1998). Isolation and characterization of a novel endogenous peptide ligand for the human APJ receptor.. Biochem Biophys Res Commun.

[36] Zeng XJ, Zhang LK, Wang HX, Lu LQ, Ma LQ (2009). Apelin protects heart against ischemia/reperfusion injury in rat.. Peptides.

[37] Kunduzova O, Alet N, esque-Touchard N, Millet L, Castan-Laurell I (2008). Apelin/APJ signaling system: a potential link between adipose tissue and endothelial angiogenic processes.. FASEB J.

[38] Eyries M, Siegfried G, Ciumas M, Montagne K, Agrapart M (2008). Hypoxia-induced apelin expression regulates endothelial cell proliferation and regenerative angiogenesis.. Circ Res.

[39] Quazi R, Palaniswamy C, Frishman WH (2009). The emerging role of apelin in cardiovascular disease and health.. Cardiol Rev.

[40] Kidoya H, Ueno M, Yamada Y, Mochizuki N, Nakata M (2008). Spatial and temporal role of the apelin/APJ system in the caliber size regulation of blood vessels during angiogenesis.. EMBO J.

[41] Nahrendorf M, Pittet MJ, Swirski FK (2010). Monocytes: protagonists of infarct inflammation and repair after myocardial infarction..

[42] Kim SW, Kim H, Yoon YS (2011). Advances in bone marrow-derived cell therapy: CD31-expressing cells as next generation cardiovascular cell therapy..

[43] Kim SW, Kim H, Cho HJ, Lee JU, Levit R (2010). Human peripheral blood-derived CD31+ cells have robust angiogenic and vasculogenic properties and are effective for treating ischemic vascular disease..

[44] Zubair AC, Malik S, Paulsen A, Ishikawa M, McCoy C (2010). Evaluation of mobilized peripheral blood CD34(+) cells from patients with severe coronary artery disease as a source of endothelial progenitor cells.. Cytotherapy.

[45] Doi H, Iso T, Shiba Y, Sato H, Yamazaki M (2009). Notch signaling regulates the differentiation of bone marrow-derived cells into smooth muscle-like cells during arterial lesion formation.. Biochem Biophys Res Commun.

